# Vitamin D status in critically ill patients: back to basics!

**DOI:** 10.1186/s13054-014-0611-0

**Published:** 2014-11-11

**Authors:** Anne-Françoise Rousseau, Etienne Cavalier

**Affiliations:** Burn Centre and General Intensive Care Department, University of Liège, University Hospital, Sart-Tilman, B-4000 Liège, Belgium; Clinical Chemistry Department, University of Liège, University Hospital, Sart-Tilman, B-4000 Liège, Belgium

The problem of vitamin D deficiency during intensive care requires a number of clarifications. Initially, it is essential to focus on the fundamentals of vitamin D status assessment. Total 25-hydroxyvitamin D (25OH-D) is currently considered as the best indicator of vitamin D status. Due to practical reasons (less knowledge needed, higher throughput), immunoassays are generally preferred to measure serum 25OH-D levels. In the critical care context, however, they may be biased due to matrix effects [1]. During the acute phase of critical care, these assays should then be abandoned in favor of liquid chromatography-tandem mass spectrometry. Moreover, standardization of vitamin D assays is crucial: such a process is ongoing worldwide but, to date, is still incomplete. It is important to note that both points are rarely considered in the published studies related to vitamin D in critically ill patients: the data may thus be interpreted with caution.

The bioavailable and free fraction of 25OH-D are attractive complementary markers of vitamin D status; however, progress is needed before establishing their evidence [2]. We need an accurate assay rather than formulas to measure free and bioavailable concentrations. We also need to determine normal or optimal ranges and to investigate their clinical impact. In the future, it seems important to consider these analytical prerequisites when studying vitamin D in critically ill patients. Meanwhile, these patients should benefit from the potential beneficial effects of vitamin D. Cholecalciferol is, in fact, a cheap medication with a wide therapeutic window. We believe that the critically ill should be supplemented with vitamin D as they are clearly at risk of deficiency. Meanwhile, for specific protocols, intensivists should at least follow the Endocrine Society guidelines [3,4].

## Abbreviation

25OH-D: 25-hydroxyvitamin D
